# Role of myeloid cells in system-level immunometabolic dysregulation during prolonged successful HIV-1 treatment

**DOI:** 10.1097/QAD.0000000000003512

**Published:** 2023-03-21

**Authors:** Sara Svensson Akusjärvi, Shuba Krishnan, Anoop T. Ambikan, Flora Mikaeloff, Sivasankaran Munusamy Ponnan, Jan Vesterbacka, Magda Lourda, Piotr Nowak, Anders Sönnerborg, Ujjwal Neogi

**Affiliations:** a Division of Clinical Microbiology, Department of Laboratory Medicine, Karolinska Institutet, ANA Futura, Campus Flemingsberg, Stockholm, Sweden; b HIV Vaccine Trials Network, Vaccine and Infectious Disease Division, Fred Hutchinson Cancer Research Centre, Seattle, USA; c Department of Medicine Huddinge (MedH), Karolinska Institutet, Stockholm; d Center for Infectious Medicine, Department of Medicine Huddinge, Karolinska Institutet, ANA Futura, Campus Flemingsberg; e Childhood Cancer Research Unit, Department of Women's and Children's Health, Karolinska Institutet, Stockholm, Sweden.

**Keywords:** flow cytometry, HIV-1, inflammatory markers, metabolomics, myeloid cells

## Abstract

Why people with HIV-1 on ART (PWH_ART_) display convoluted metabolism and immune cell functions during prolonged suppressive therapy is not well evaluated. In this study, we aimed to address this question using multiomics methodologies to investigate immunological and metabolic differences between PWH_ART_ and HIV-1 negative individuals (HC).

**Design:**

Cross-sectional study

**Methods:**

Untargeted and targeted metabolomics was performed using gas and liquid chromatography/mass spectrometry, and targeted proteomics using Olink inflammation panel on plasma samples. The cellular metabolic state was further investigated using flow cytometry and intracellular metabolic measurement in single-cell populations isolated by EasySep cell isolation. Finally, flow cytometry was performed for deep-immunophenotyping of mononuclear phagocytes.

**Results:**

We detected increased levels of glutamate, lactate, and pyruvate by plasma metabolomics and increased inflammatory markers (e.g. CCL20 and CCL7) in PWH_ART_ compared to HC. The metabolite transporter detection by flow cytometry in T cells and monocytes indicated an increased expression of glucose transporter 1 (Glut1) and monocarboxylate transporter 1 (MCT-1) in PWH_ART_. Single cell-type metabolite measurement identified decreased glucose, glutamate, and lactate in monocytic cell populations in PWH_ART_. Deep-immunophenotyping of myeloid cell lineages subpopulations showed no difference in cell frequency, but expression levels of CCR5 were increased on classical monocytes and some dendritic cells.

**Conclusions:**

Our data thus suggest that the myeloid cell populations potentially contribute significantly to the modulated metabolic environment during suppressive HIV-1 infection.

## Introduction

Successful long-term antiretroviral therapy (ART) inhibits human immunodeficiency virus type-1 (HIV-1) replication for a prolonged period in people with HIV-1 (PWH). We and others have earlier reported metabolic modifications involving dysregulation of the amino acid (AA) and central carbon metabolism in PWH on successful long-term ART (PWH_ART_) [1–9]. Our recent study also reported a system-level upregulation of oxidative phosphorylation (OXPHOS) and glycolysis in PWH_ART_ compared to HIV-1 positive elite controllers who naturally control viral replication [10]. These alterations can play a role in latent reservoir dynamics and immunosenescence in PWH_ART_. In our earlier studies from India [1], Denmark [2], and Cameroon [3], we reported persistent metabolic reprogramming in patients with successful therapy. A subset of these PWH_ART_ also showed an altered metabolic profile linked with the development of age-related comorbidities that can potentiate accentuated aging or age-related diseases.

In this study, we performed a system-level plasma metabolomics profiling using untargeted and targeted metabolomics in a well defined Swedish cohort of PWH_ART_, together with matched HIV-negative controls (HC). We also performed immune phenotyping of metabolite transporters for some critical metabolites altered in PWH_ART_ and intercellular measurements of the key metabolites in different cell populations. Finally, we phenotyped myeloid cell lineages, focusing on the expression levels of chemokine receptors that play a significant role in cellular trafficking during inflammation. We observed that intercellular metabolites and metabolite transporters are altered in monocytes but not T cells, potentially linked with the systemic metabolic environment. Our study thus provides a comprehensive understanding of immuno-metabolic regulation during long-term successful therapy.

## Methods

### Cohort description

This study included one group of HIV-1 infected individuals with suppressed viremia (PWH_ART_, *n* = 64), one group with viremia (PWH_VP_, *n* = 24), and HIV-1-negative controls (HC, *n* = 37) (total cohort characteristics can be viewed in Table 1, Supplemental Digital Content). The median suppressive treatment of PWH_ART_ was 7 years [interquartile range (IQR) 6–13]. We also included paired samples between PWH_VP_ (*n* = 11) and PWH_ART_ (*n* = 11) after a median of 8 years (IQR 6–8 years) of suppressive therapy. Clinical parameters for the longitudinal cohort can be viewed in Table 2, Supplemental Digital Content. In Table 3, Supplemental Digital Content number of samples used per experimental method is specified. The HC samples were collected at the Karolinska University Hospital, Huddinge, and the inclusion criteria were no known conditions or infections at the time of sampling.

The study was approved by the Regional Ethics Committee of Stockholm and performed according to the Declaration of Helsinki. All participants gave informed consent before inclusion in the study.

### Metabolomics

Plasma un-targeted metabolomics was performed using the HD4 Platform (Metabolon, Morrisville, North Carolina, USA) in a cohort including HC (*n* = 22), PWH_ART_ (*n* = 29), and PWH_VP_ (*n* = 11). The samples were selected randomly. The method was performed as previously described [11]. Plasma targeted metabolomics was performed towards central carbon metabolism (CCM) and sugars using gas chromatography–mass spectrometry (GC–MS) and amino acids by liquid chromatography (LC)–MS/MS in a cohort of HC (*n* *=* 37), PWH_ART_ (*n* *=* 55), and PWH_VP_ (*n* *=* 24) at the Swedish Metabolomics Centre (Umeå, Sweden).

### Proteomics

Targeted plasma proteomics was performed using the Olink inflammation panel (Olink, Sweden) as previously described [12]. In brief, a proximity extension assay was used to evaluate the plasma levels of a set panel of proteins. Cycle threshold (Ct) values were normalized to an extension control and inter-plate control, and all proteins were reported as normalized protein expression levels (NPX).

### Bioinformatic analysis

Differential enrichment analysis was performed using R package limma v3.50.0 [13]. Mann–Whitney *U* test was performed using R package stats v4.1.2. Heatmaps were created using R package ComplexHeatmap v2.10.0 [14]. UMAP dimensionality reduction was done with R package umap v2.7.0. Boxplots, bar plots, and bubble plots were generated using the R package ggplot2 v3.3.5/. Network and community analysis was performed as previously described by us [15].

### Isolation of CD4^+^ T cells and monocytes

Cell subpopulations of CD4^+^ T cells (CD3^+^CD4^+^) and monocytes (CD3^−^CD14^+^) were isolated using EasySep magnets (STEMCELL Technologies, Vancouver, British Columbia, Canada). First, PBMCs were washed in FACS buffer (PBS + 2% FBS + 2 mmol/l EDTA) and treated with DNAse (100 ug/ml) (StemCell, #7900) for 15 min at room temperature. Subsequently, Easysep Human CD4^+^ positive selection kit II (StemCell, #17852) was used according to the manufacturer's protocol, and the flowthrough used for Easysep Human monocyte isolation kit (StemCell, #19359), according to the manufacturer's protocol.

### Intracellular metabolite detection

From isolated CD4^+^ T cells and monocytes, 30 000 cells were used for metabolite detection in duplicates for each sample. Lactate-Glo assay (Promega, #J5022; Promega, Madison, Wisconsin, USA), Glutamate-Glo assay (Promega, #J7022), and Glucose-Glo assay (Promega, #J6022) were used to measure intracellular metabolites according to the manufacturer's instructions. Luminescence was measured using Varioskan microplate reader (ThermoFisher Scientific, Waltham, Massachusetts, USA).

### Flow cytometry

Purity evaluation of isolated cell populations was acquired on BD Fortessa (BDBiosciences, Franklin Lakes, New Jersey, USA). Expression levels of metabolite transporters was acquired on BD Symphony (BdBiosciences). The myeloid lineage panel was acquired on BD Symphony (BdBiosciences). Staining was complemented with Dead cell (Near-IR or Aqua) viability stain (Invitrogen, ThermoFisher, Scientific). Specifics about antibodies can be found in Table 4, Supplemental Digital Content. All flow cytometry analysis was performed using FlowJo 10.8.1 (TreeStar Inc., Ashland, Oregon, USA).

### Statistics

Statistical analysis was performed using Mann−Whitney *U*-test in Prism 9.3.0 (GraphPad, Software, San Diego, California, USA) or Rstudio (v.1.3.1056; R Foundation for Statistical Computing, Vienna, Austria). In omics data, the multiple hypothesis corrections were performed using Benjamini−Hochberg (BH) method.

## Results

### Increased levels of glutamate, pyruvate and lactate in people with HIV-1 on antiretroviral therapy compared to HIV-negative controls

**Fig. 1:**
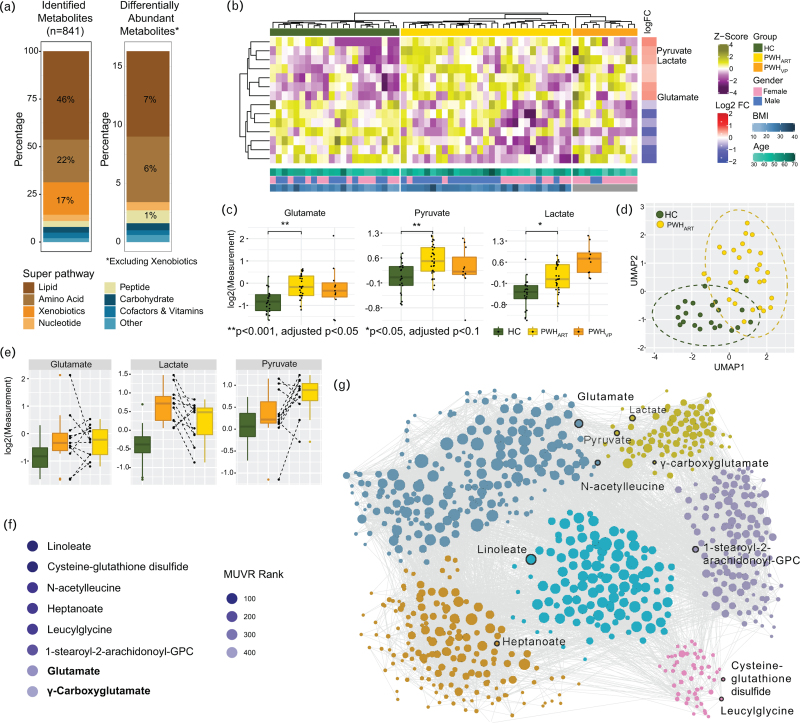
Untargeted metabolomics showed that PWH_ART_ have increased levels of glutamate, pyruvate, and lactate.

We performed untargeted metabolomic analysis using the HD4 Platform (Metabolon) on plasma samples obtained from PWH_ART_ (*n* *=* 29), untreated PWH with viremia (PWH_VP_, *n* *=* 11), and matched HIV-negative controls (HC, *n* *=* 22). A total of 841 metabolites were detected, of which 143 (17%), belonging to xenobiotics, were excluded from further analysis as these are not naturally produced (Fig. 1a). After adjusting for gender, body mass index (BMI), and age, known to influence the plasma metabolite profile, 14 metabolites were significantly different (adjusted *P* < 0.1) between PWH_ART_ and HC (Fig. 1a and Supplementary File 1, Supplemental Digital Content). We also detected 36 (adjusted *P* < 0.1) metabolites that differed between PWH_VP_ and PWH_ART_ and 70 (adjusted *P* < 0.1) between PWH_VP_ and HC. Therefore, a pronounced host metabolic alteration was detected during the initial viraemic phase. Since our primary aim was to identify the metabolic dysregulation in PWH_ART_ compared to matched HC, we restricted the analysis to these two groups. Heatmap visualization of significantly different metabolites between PWH_ART_ and HC showed a distinct enrichment pattern of metabolites between the groups (Fig. 1b). This included a significant enrichment of glutamate, pyruvate, and lactate in PWH_ART_ (Fig. 1c). Uniform manifold approximation and projection (UMAP) could clearly distinguish clusters for PWH_ART_ and HC based on these three metabolites (Fig. 1d). For 11 individuals, we had paired samples from the initial viraemic phase without therapy and after a median duration of eight years of treatment. In five of these patients, the median levels of glutamate, both before and during treatment, were higher than in HC, while glutamate levels increased following treatment initiation. In most of the patients, lactate levels decreased (9 of 11) while pyruvate levels increased (7 of 11) following therapy (Fig. 1e). Next, we tried to identify biomarkers that could differentiate HC from successfully treated PWH_ART_ using multivariate methods with unbiased variable selection in R (MUVR). This analysis identified glutamate and γ-carboxyglutamate as key biomarkers (Fig. 1f). We further performed weighted co-expression analysis using the Leiden algorithm, where coordination between pyruvate, lactate, and glutamate was observed (Fig. 1g). Therefore, our data indicate a dysregulation of glutamate metabolism in PWH_ART_ with long-term successful HIV-1 treatment. This modulation could play a significant coordinating role in pyruvate and lactate metabolism.

(a) Proportion of total metabolites identified and differentially abundant metabolites between HC and PWH_ART_ from untargeted metabolomics data. Xenobiotic metabolites were excluded from both datasets. (b) Heatmap showing metabolite abundance pattern of significantly changed metabolites in PWH_ART_ compared to HC (adj. *P* < 0.1). Column annotation represents the study cohort, and the bottom annotation includes the age, gender, and BMI of each patient. Log FC representation denotes log2 scaled fold change in PWH_ART_ compared to HC. (c) Boxplot of glutamate, pyruvate, and lactate levels in HC, PWH_ART,_ and PWH_VP_. (d**)** Sample distribution based on significantly changed metabolites between HC and PWH_ART_ using UMAP dimensionality reduction. Ellipses denote 95% confidence interval. (e) Boxplot showing glutamate, pyruvate, and lactate levels in longitudinal metabolomics data. Paired samples are connected by the dotted line. (f) Metabolites were identified as biomarkers of PWH_ART_ compared with HC using MUVR biomarker prediction. Color gradient of bubbles is relative to MUVR rank. (g) Weighted co-expression analysis using Leiden algorithm and represented as a network. Metabolites identified as predicted biomarkers from F and dysregulated metabolites identified from B are labelled. Statistical significance was determined using Mann−Whitney *U*-test (*P* *<* 0.05). HC, HIV-1 negative controls; MUVR, multivariate methods with unbiased variable selection in R; PWH_ART_, people with HIV on antiretroviral therapy; UMAP, uniform manifold approximation and projection.

### Dysregulated central carbon and amino acid metabolism together with elevated levels of inflammatory markers in people with HIV-1 on antiretroviral therapy

**Fig. 2:**
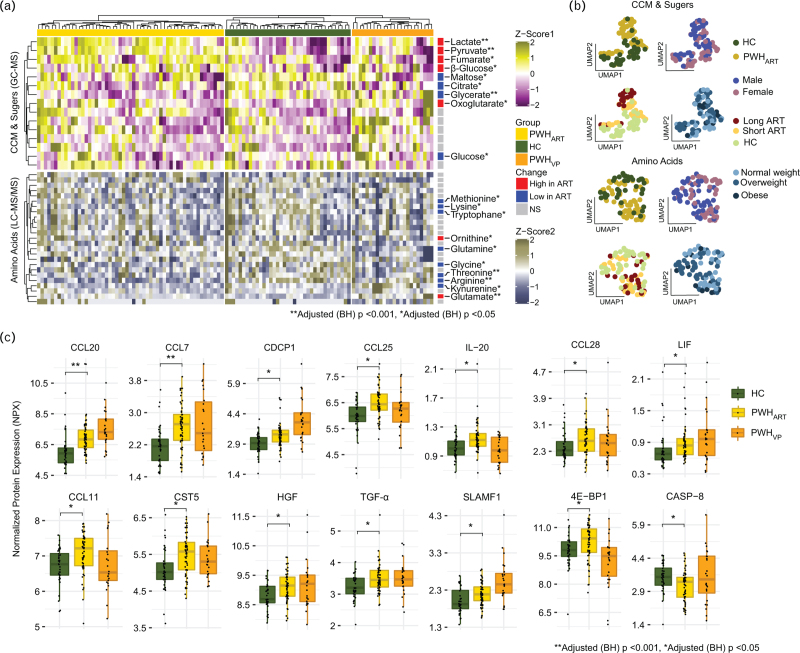
Dysregulated central carbon and amino acid metabolism together with elevated levels of inflammatory markers in PWH_ART_.

Next, to validate our findings in a larger cohort of PWH_ART_ (*n* *=* 55), PWH_VP_ (*n* *=* 24), and HC (*n* *=* 37), we performed targeted metabolomic analysis for AA and metabolites of the central carbon metabolism (CCM) and sugars. Among the quantified AA (*n* *=* 26), CCM metabolites, and sugars (*n* *=* 15), 19 metabolites were significantly different (adjusted *P* < 0.05) between PWH_ART_ and HC (Fig. 2a). Of these, the majority of AA (tryptophan, methionine, lysine, glutamine, glycine, arginine, threonine, and kynurenine) decreased in PWH_ART_ while glutamate was increased (Fig. 2a). Amongst the CMM metabolites and sugars, we detected a decrease in glucose while its isoform beta-glucose was increased in PWH. Furthermore, a similar trend with an increase in lactate and pyruvate was seen for the untargeted metabolomics, strengthening the result (Fig. 2a). As the proportion of metabolites can be a consequence of clinical parameters or confounders, we next employed UMAP to evaluate if gender, HIV-positive status, BMI, or length of treatment could separate PWH_ART_ from HC. The UMAP analysis identified that alterations in CCM metabolites and sugars were associated with the duration of therapy (Fig. 2b). Finally, to evaluate the inflammation level, we performed secretome analysis in plasma using the Olink Inflammation panel. Of the 92 proteins identified, C−C motif chemokine ligand 20 [CCL20/macrophage inflammatory protein 3 alpha (MIP-3α)] and CCL7 [monocyte chemotactic protein 3 (MCP-3)] were significantly higher (adjusted *P* < 0.001) in PWH_ART_ compared to HC (Fig. 2c). Another 12 inflammatory markers were significantly higher in PWH_ART_ than HC, using a significance cut-off adjusted *P* < 0.05. These markers included CCL25 [thymus expressed chemokine (TECK)], CCL28 [mucosa-associated epithelial chemokine (MEC)], CCL11 (eotaxin-1), cystatin-D (CST5), interleukin 20 (IL-20), signaling lymphocytic activation molecule 1 (SLAMF1), CUB domain-containing protein 1 (CDCP1), eukaryotic translation initiation factor 4E-binding protein 1 (4EBP1), transforming growth factor-alpha (TGF-α), leukemia inhibitory factor (LIF), and hepatocyte growth factor (HGF) (Fig. 2c). Only caspase-8 (CASP-8) showed a significant decrease in PWH_ART_ compared to HC. Thus, our data suggest that PWH_ART_ has a dysregulated energy metabolism, in AA, CCM, and sugars, together with increased markers of inflammation like MIP-3α or MCP-3.

(a) Heatmap showing metabolite abundance pattern of targeted metabolomics data. Normalized levels of central carbon metabolism (CCM), sugar metabolites and amino acids are shown from the cohorts. Column annotation denotes the study cohorts and right annotation shows the metabolite levels in PWH_ART_ compared to HC. Significantly changed metabolites in PWH_ART_ compared to HC are labelled. (b) Sample distribution based on targeted metabolomics data of CCM, sugar metabolites and amino acids by UMAP dimensionality reduction. Individual UMAPs are coloured in respective category for cohort, gender, duration of treatment, and BMI. (c) Boxplot of significantly regulated proteins in PWH_ART_ compared to HC (adj.*P* < 0.05). Statistical significance was determined using Mann−Whitney *U*-test (adjusted *P* *<* 0.05). HC, HIV-1 negative controls; PWH_ART_, people with HIV on antiretroviral therapy; UMAP, uniform manifold approximation and projection.

### Intracellular metabolites and transport are dysregulated in monocytes during suppressive HIV-1 infection

**Fig. 3:**
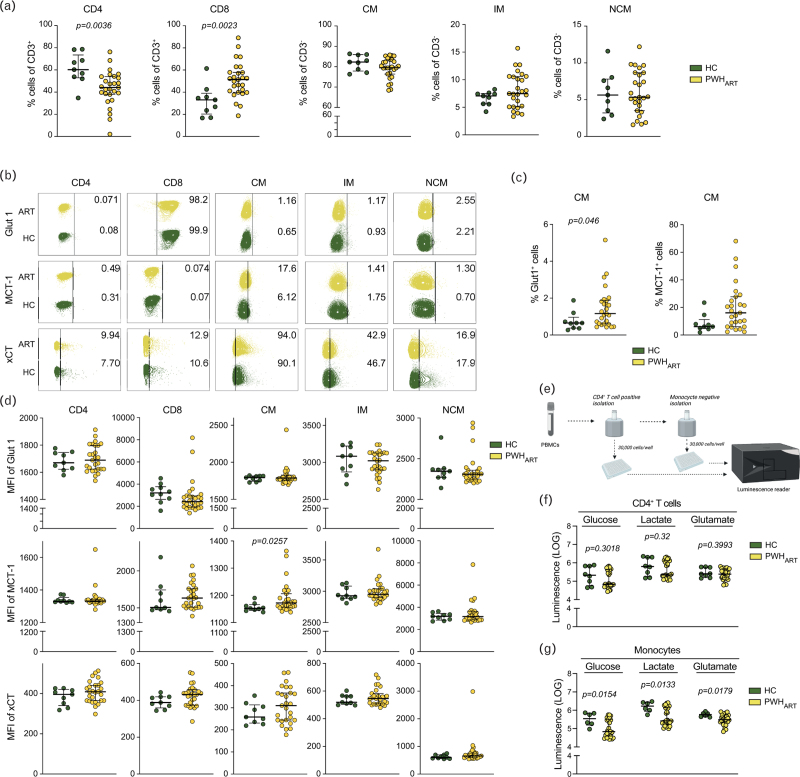
Intracellular metabolites and transport are dysregulated in monocytes during suppressive HIV-1 infection.

Viral infections hijack the cellular metabolic environment, mostly AAs, CCM, and sugars, for their support and propagation [11,16,17]. As the significant systemic alteration of the metabolites was glucose, pyruvate/lactate, and glutamate, next we performed flow cytometry analysis of metabolite transporters, glucose transporter-1 (Glut1), pyruvate and lactate transporter monocarboxylate transporter 1 (MCT-1), and cysteine/glutamate antiporter (xCT) in a cohort of HC (*n* *=* 9) and PWH_ART_ (*n* *=* 27) (Fig. 1A, Supplemental Digital Content). The CD4^+^ T cells were decreased while CD8^+^ T cells were increased in PWH_ART_ compared to HC (Fig. 3a). No significant differences were observed in classical (CM), intermediate (IM), or nonclassical monocytes (NCM). Receptor expression analysis showed that the percentage of CM^+^Glut1^+^ increased in PWH_ART_ compared to HC (Fig. 3b and c). Analysis of the median fluorescence intensity (MFI) showed no difference in CD4^+^ or CD8^+^ T cells (Fig. 3d). Even though there was no significant difference in MCT-1^+^ CM, the MFI of MCT-1 was increased on CM in PWH_ART_ compared to HC (Fig. 3c and d). To further evaluate the intracellular metabolite levels in blood cell populations, a cohort of HC (*n* *=* 10) and PWH_ART_ (*n* *=* 29) was used to isolate CD4^+^ T cells and monocytes (Fig. 3e and Fig. 1B, Supplemental Digital Content). One PWH_ART_ sample was excluded due to low cell viability. After CD4^+^ T-cell isolation, two HC samples were excluded from the analysis due to low cell count. The purity of isolated CD4^+^ T cells had a median of 97% (IQR 93.9–97.575) (Fig. 1C, Supplemental Digital Content). Intracellular measurement of glucose, lactate, and glutamate showed no significant difference within the CD4^+^ T-cell subset (Fig. 3f). After monocyte isolation, four HC samples and one PWH_ART_ sample were excluded from the analysis due to low cell count. The purity of the monocytes had a median of 93.15% [interquartile range (IQR) 95.55–90.55] (Fig. 1D, Supplemental Digital Content). Intracellular measurement of glucose, lactate, and glutamate showed a significant decrease of all three metabolites in PWH_ART_ compared to HC (Fig. 3g). These data indicate that the main differences in the metabolic profile occur in the monocytes during long-term suppressive therapy.

Metabolite uptake and secretion in HC (*n* *=* 9) and PWH_ART_ (*n* *=* 27). (a) Proportion of CD4^+^ and CD8^+^ T cells and classical (CM), intermediate (IM), and nonclassical (NCM) monocytes in PBMCs. (b) Contour plots showing percentage of cells expressing Glut1, MCT-1, and xCT in CD4^+^, CD8^+^, CM, IM, and NCM. The contour plots show a representing sample in each group with the median expression level within that group. (c) Percentage of CM expressing Glut1 or MCT-1 PWH_ART_ and HC. (d) Median fluorescence intensity (MFI) of Glut1, MCT-1, and xCT expression on CD4^+^, CD8^+^, CM, IM, and NCM cell populations in HC and PWH_ART_. (e) Schematic representation of CD4^+^ and monocyte isolation for intracellular metabolite measurement. (f) Intracellular measurement of glucose, lactate, and glutamate in a cohort of HC (*n* *=* 8) and PWH_ART_ (*n* *=* 28) in CD4^+^ T cells. (g) Intracellular measurement of glucose, lactate, and glutamate in a cohort of HC (*n* *=* 6) and PWH_ART_ (*n* *=* 27) in monocytes. Statistical significance was evaluated using Mann−Whitney *U*-test (*P* *<* 0.05) and represented as median with interquartile range (IQR). See also Figure S1. HC, HIV-1 negative controls; IQR, interquartile range; MUVR, multivariate methods with unbiased variable selection in R; PWH_ART_, people with HIV on antiretroviral therapy; UMAP, uniform manifold approximation and projection.

### Characterization of monocytic subpopulations in people with HIV-1 on antiretroviral therapy vs. HIV-1 negative controls

**Fig. 4:**
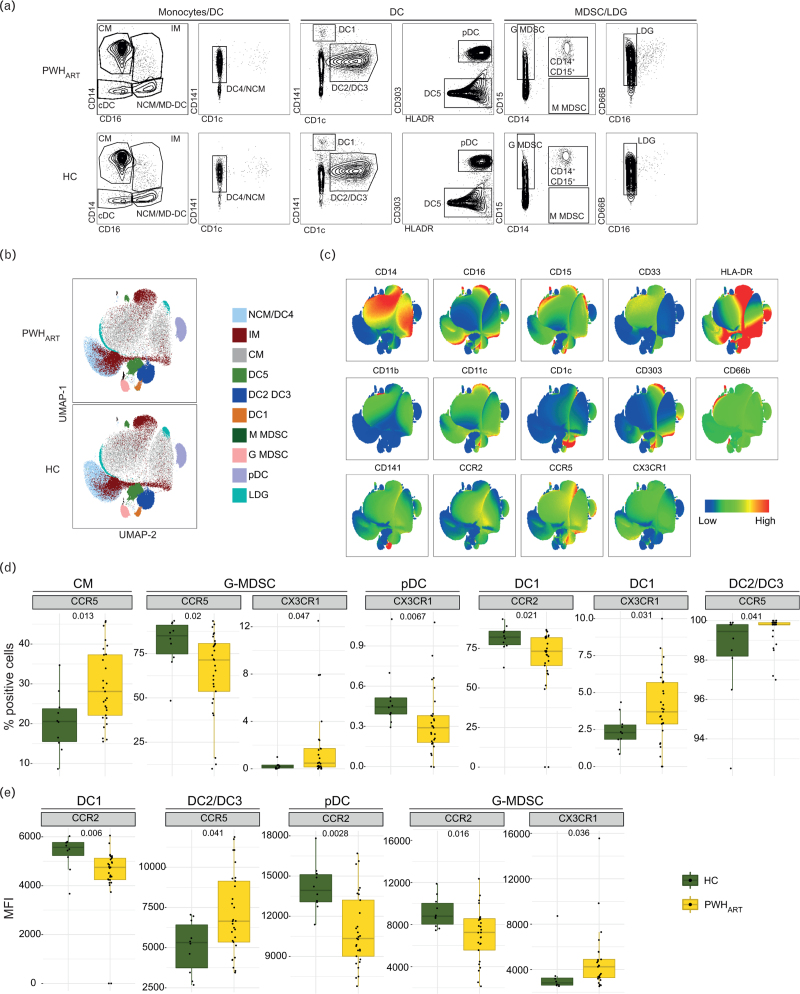
Receptor expression of CCR2, CCR5, and CX3CR1 on myeloid cell subsets differentiates PWH_ART_ from HC.

As we identified the main differences in intracellular metabolite and transporter expression in monocytes, we wanted to evaluate the distribution in monocytic cell populations. Therefore, we ran a panel characterizing myeloid cells in HC (*n* *=* *10*) and PWH_ART_ (*n* *=* 29) (Fig. 2A, Supplemental Digital Content). The panel identified subsets of monocytes, dendritic cells (DCs), and myeloid-derived suppressor cells (MDSCs) (Fig. 4a). The distribution of cell populations can be viewed in Fig. 4b. Even as the proportion of DC2/DC3 showed a slight reduction in PWH_ART_ (*P* *=* 0.085), no significant differences were detected in the frequency of cell populations except for the frequency of CD11b^+^CD33^+^ low density granulocytes (LDGs) (Fig. 2B, Supplemental Digital Content). Additionally, we looked at chemokine receptor expression CCR2, CCR5, and CX3CR1 as they are known markers of migration and activation of myeloid lineages (Fig. 4c). CM expressing CCR5 were increased in frequency, while CCR5^+^ granulocytic (G)-MDSC were decreased and CX3CR1^+^ G-MDSC were increased in PWH_ART_ compared to HC (Fig. 4d). In DC lineages, the frequency of CX3CR1^+^ plasmacytoid (p)DC and CCR2^+^ DC1 was decreased, while the frequency of CX3CR1^+^ DC1 and CCR5^+^ DC2/DC3 was increased in PWH_ART_ compared to HC (Fig. 4d). No other significant differences in the proportion of cells expressing any of the receptors were identified between the groups (Fig. 3A, Supplemental Digital Content). Within the cell populations, the MFI was decreased for CCR2 on DC1, pDC, and G-MDSC in PWH_ART_ compared to HC (Fig. 4E). Furthermore, an increase in MFI for CCR5 on DC2/DC3 and CX3CR1 on G-MDSC was observed in PWH_ART_ compared to HC (Fig. 4e and Fig. 3B, Supplemental Digital Content). Collectively, this data shows the relevance of chemokine receptors on myeloid lineages as the key regulators of myeloid cell trafficking during HIV-1 infection, rather than the distribution of cell populations during suppressive therapy.

Characterization of myeloid lineages in HC (*n* *=* 10) and PWH_ART_ (*n* *=* 29). (a) Gating defines all cell populations identified in the cohort. (b) UMAP representation of the distribution of classical monocytes (CM), intermediate monocytes (IM), nonclassical monocytes/dendritic cell 4 (NCM/DC4), dendritic cell 1 (DC1), dendritic cell 2/3 (DC2/DC3), dendritic cell 5 (DC5), plasmacytoid dendritic cells (pDC), granulocyte-like myeloid suppressor cells (G-MDSC), monocytic myeloid-derived suppressor cells (M-MDSC), and low-density granulocytes (LDG) in PWH_ART_ and HC. (c) Heatmap representation of each marker used in the UMAP. (d) Frequency (%) of cells expressing CCR5 (CM, G-MDSC, and DC2/DC3), CCR2 (DC1), and CX3CR1 (G-MDSC, pDC, and DC1). (e) Median fluorescence intensity (MFI) of CCR2 (DC1, pDC, and G-MDSC), CCR5 (DC2/DC3), and CX3CR1 (G-MDSC) on the indicated cells. Statistical significance was determined using Mann−Whitney *U*-test (*P* *<* 0.05) and represented with median and IQR. See also Figure S2 and Figure S3. HC, HIV-1 negative controls; IQR, interquartile range; MUVR, multivariate methods with unbiased variable selection in R; PWH_ART_, people with HIV on antiretroviral therapy; UMAP, uniform manifold approximation and projection.

## Discussion

Our comprehensive immuno-metabolic study in PWH_ART_ identified system-level alterations in amino acid metabolism during long-term treatment. We identified a coordinated role of pyruvate, glutamate, and lactate in HIV-1 infection, indicating central carbon and energy metabolism dysregulation. Furthermore, the plasma inflammatory markers MIP-3α and MCP-3 increased in PWH_ART_. Macrophages and monocytes mainly produce the MIP-3α, while MCP-3 is a chemoattractant that plays a significant role in monocyte mobilization and trafficking to the inflammation sites. Therefore, our data indicate that monocytes play a potential role in the modulation of the inflammatory profile during suppressive ART. This could also be linked with the metabolic alterations in the monocytic subsets for transporter expression and intracellular levels. Finally, our deep immune profiling of myeloid cell lineages indicated an altered expression of CCR5 and CCR2 in monocytes and subsets of DCs. This data further points towards the role of myeloid cell lineages in cell trafficking, regulation, and persistent inflammation in PWH during successful ART.

The three main pathways of CCM and energy metabolism are compartmentalized between the cytoplasm and mitochondria, namely, glycolysis/gluconeogenesis, glutamine metabolism (glutaminolysis), and tricarboxylic acid cycle (TCA cycle) [providing electrons for OXPHOS]. The host dependencies for HIV-1 include glycolysis and TCA [18]. Susceptibility to HIV-1 is also regulated by a cell's metabolic activity and activation stage, where CD4^+^ T cells with elevated OXPHOS and glycolysis are more permissive to infection [9,19]. In this study, we detected decreased Glut1 expression on CD8^+^ T cells while the percentage of CM expressing the receptor was increased. The uptake of glucose through Glut1 is essential for viral production [20]. In our recent study, we detected reduced Glut1 expression on T-lymphocytes in HIV-positive elite controllers compared to the HIV-negative controls, which could be one mechanism restricting HIV-1 infection [21]. A recent study also indicated that latent HIV-1 reservoirs use glutaminolysis as an alternative fuel source for energy generation [22]. Our cohort detected decreased glutamine and increased glutamate in plasma from PWH_ART_. Glutaminolysis is the primary pathway charging the TCA-cycle and OXPHOS in naive and memory T-cell subsets. Higher HIV-1 infections have been shown in T cells selected for OXPHOS activity [19] and compromised metabolic steps preceding OXPHOS result in lipid accumulation [22]. Furthermore, we recently observed how modulation of glutaminolysis significantly affects the reactivation of the latent virus [3]. This hints toward a substantial role of glutaminolysis in HIV-1 reactivation.

A recent seminal study also showed that senescent cells relied on glutaminolysis and proposed that inhibiting glutaminolysis in the aging body could prevent age-associated disorders and even prolong the lifespan [23]. It is known that in glutamate toxicities, pyruvate plays a significant role in quenching glutamate to keep the level of inflammation low [24]. Our longitudinal data thus indicates that the increase of pyruvate could result from elevated glutamate levels. In our earlier cross-sectional studies, we observed higher glutamate levels in PWH_ART_ in cohorts from India [1], Cameroon [3], and Denmark [2]. These cohorts, collectively including more than 500 PWH, indicated a prevalent disruption of glutaminolysis, that is, lysis of glutamine to glutamate, in PWH_ART_. We also saw that glutaminolysis was central to comorbidities such as metabolic syndrome (MetS) [2,3]. Interestingly, the level of pyruvate was high in the Danish cohort but not in India or Cameroon. Additionally, the level of inflammation was high in the Indian cohort [25], while our earlier Swedish study on long-term treated individuals (nearly two decades) indicated near normalization of the inflammatory profile [26]. Based on these observations, we posit that the increased level of pyruvate in cohorts from high-income countries could be linked with the low level of persistent inflammation. This alteration could be associated with using newer antiretrovirals with a better toxicity profile while indicating an enhanced metabolic profile and improved quality of life.

Herein, we also show how intracellular metabolic modulation mainly occurs in the monocytic cell population in PWH_ART_. We did not see any differences in the frequency of myeloid cell populations, indicative of maintained cellular frequencies during suppressive ART. However, there was a variation in the receptor expression of CCR2, CCR5, and CX3CR1 in some sub-populations. Activation of these chemokines’ receptors mediates immune cell trafficking. Even as the general classification of CM is CCR2^high^ and CX3CR1^low^, our cohort exhibited an increase in CCR5 in PWH_ART_[27]. CCR5 is one of the main co-receptors used for HIV-1 entry into cells, and in monocytes, the dependency of CCR5 for the M-tropic virus is well described [28]. Earlier studies have also shown how activated monocytes and macrophages shift to a glycolytic metabolism by increasing the expression of Glut1, one of the primary glucose transporters [29]. As described here by us and others, these activated Glut1^+^ monocytes were enriched during HIV-1 infection [30]. Therefore, the elevated levels of CCR5 on CM could indicate an increased susceptibility towards HIV-1 or, on a phenotype level, an increased transition into a proinflammatory phenotype to elicit heightened inflammatory responses. Simultaneously, the evaluated immune activation seen in PWH_ART_ could support an environment of increased glucose metabolism and higher activation-induced differentiation of monocytes, as previously reported in T cells [31,32].

Even as a complete characterization of the effect of HIV-1 infection on DC subsets and progenitors is limited, several studies have shown the depletion of DCs during acute viral infections (e.g. COVID-19 [33] and Hantavirus [34]). Possibly, similar events occur during initial HIV-1 viremia but normalize during suppressive ART. This would be in accordance with our data, where we did not see any differences in the frequency of DC subsets. However, our data show that the main differences in receptor expression occur in the DCs with a decrease of CCR2 and CX3CR1 in some subpopulations, while CCR5 increases in DC2/DC3. The DCs belong to the mononuclear phagocytes (MNPs) together with monocytes and macrophages, which are crucial for linking the innate and adaptive immune systems [35]. In HIV-1, DCs can transfer the virus to T cells by delivery of membrane-bound virus through a viral synapse or *de novo* synthesis of virus from infected cells [35]. After capturing the virus and antigen presentation, DCs migrate to the lymph nodes and spleen. This can facilitate HIV-1 spread to compartments rich in target cells for infection. However, our data do not indicate increased dissemination of DC subsets to distant body compartments as the frequencies of DC subsets are maintained during suppressive ART [36]. Instead, the altered chemokine receptor expression could hint at the function of immune cells in PWH_ART_. For the CX3CR1 receptor, also known as the fractalkine receptor, the receptor-ligand interaction is strongly linked to the survival of immune cells [37]. Thus, the reduced expression in some DC subsets in PWH_ART_ could indicate a decreased capacity to counteract cell death mechanisms. This could result from the hypothesized rapid DC decline during peak viremia. MNPs are potent producers of CCL2, the ligand for CCR2, mediating a proinflammatory function [38]. A reduced expression of CCR2 could affect the responsiveness of DC recruitment to the site of inflammation. Our results show a dysregulated phenotype on some DC subsets in PWH_ART_. However, the implication on immune cell function, metabolic reprogramming, and immunological aging during suppressive ART remain to be elucidated.

Though our study used a relatively large number of samples, including the longitudinal samples, our study has limitations that merit comments. First, the study's cross-sectional nature limits the observations to associations that do not infer causation. Second, the ART regimens were heterogeneous. Third, though we have a more homogenous group, CMV status, diet and lifestyle can influence the inflammatory status. We have initiated a large study to understand the myeloid cell dysfunction in PWH_ART._ Finally, our analysis is more steady-state in the absence of functional assays.

In conclusion, our cross-sectional study provides system-level snapshots of the metabolic reprogramming in the PWH on successful therapy, dynamics of the metabolic alterations in the longitudinal study, and its association with systemic inflammation and myeloid lineage cells. This metabolic reprogramming and altered chemokine signaling may subsequently affect monocyte trafficking and polarization during persistent inflammation. In turn, these aberrations might influence adaptive immunity. A better understating of immune cell trafficking and polarization of myeloid cell lineage in PWH on successful therapy, together with strategies to modulate the activation of macrophage phenotypes, could provide adjunctive therapeutic targets to improve metabolic health while mediating the control of viral replication in PWH.

## Acknowledgements

Author contributions: U.N conceived the study; A.S. and P.N. designed and responsible for the clinical cohorts, U.N., S.S.A., S.M.P., and S.K. planned the experiments; S.S.A. and S.K. performed the research; A.T.A. and F.M. performed the bioinformatics analysis; J.V., P.N., and A.S. contributed with patient material and interpretation of the clinical findings; S.M.P. and M.L. contributed with analysis; U.N., S.S.A., and S.K. analysed the data; U.N. and S.S.A. wrote the manuscript. All authors reviewed and critically revised the manuscript. Authors would like to thank Dr Soham Gupta, Assistant Professor Karolinska Institute, for his intellectual input in the study.

Data availability. The untargeted and targeted metabolomics and OlinkTM proteomics data is available DOI: 10.6084/m9.figshare.19589365 (upon acceptance). During the review process data can be obtained through https://figshare.com/s/f402e955f3308f4dff86.

Funding: This study was funded by the Swedish Research Council Interdisciplinary Grant 2018-06156 to UN. The authors acknowledge the support received from the Swedish Research Council grants, 2017-01330 and 2021-01756 to U.N and 2017-05848 to A.S. M.L. was supported by the Swedish Childhood Cancer Fund (TJ20180128).

### Conflicts of interest

There are no conflicts of interest.

**Posted history:** This manuscript was previously posted on Research Square: doi: https://doi.org/10.21203/rs.3.rs-1574216/v1

Supplementary File 1. Differential metabolite abundance between the groups in the untargeted metabolomics.

## Supplementary Material

Supplemental Digital Content
