# Supplementary material for: Role of myeloid cells in system-level immunometabolic dysregulation during prolonged successful HIV-1 treatment
Source: AIDS. 2023 Mar 21;37(7):1023–33. doi: 10.1097/QAD.0000000000003512 (PMC10155691; doi:10.1097/QAD.0000000000003512)
Supplement: Supplemental Digital Content [file aids-37-1023-s002.doc]

**Supplementary material**

**Role of myeloid cells in system-level immunometabolic dysregulation during prolonged successful HIV-1 treatment**

Sara Svensson Akusjärvi^1^,*, Shuba Krishnan^1^, Anoop T Ambikan^1^, Flora Mikaeloff^1^, Sivasankaran Munusamy Ponnan^2^, Jan Vesterbacka^3^, Magda Lourda^4,5^, Piotr Nowak^1,3^, Anders Sönnerborg^1,3^, and Ujjwal Neogi^1^*

^1^Division of Clinical Microbiology, Department of Laboratory Medicine, Karolinska Institutet, ANA Futura, Campus Flemingsberg, Stockholm, Sweden

^2^HIV Vaccine Trials Network, Vaccine and Infectious Disease Division, Fred Hutchinson Cancer Research Centre, Seattle, USA

^3^Department of Medicine Huddinge (MedH), Karolinska Institutet, ANA Futura, Campus Flemingsberg, Stockholm, Sweden

^4^Center for Infectious Medicine, Department of Medicine Huddinge, Karolinska Institutet, ANA Futura, Campus Flemingsberg, Stockholm, Sweden

^5^Childhood Cancer Research Unit, Department of Women’s and Children’s Health, Karolinska Institutet, Stockholm, Sweden

*Corresponding authors: Sara Svensson Akusjärvi ([sara.svensson.akusjarvi@ki.se](mailto:sara.svensson.akusjarvi@ki.se)); Ujjwal Neogi ([ujjwal.neogi@ki.se](mailto:ujjwal.neogi@ki.se))

**Supplementary Table 1: Clinical characteristics**

| **Parameter** | **PLWH_ART_** | **PLWH_VP_** | **HC** | **P-value** |
| --- | --- | --- | --- | --- |
| *N* | 64 | 24 | 37 | ND |
| Gender, Female, *N* (%) | 20 (31.2) | 11 (45.8) | 23 (62.2) | 0.01^+^ |
| **At sampling** |  |  |  | ND |
| Age in years, mean (SD) | 50 (9.3) | 48 (13.2) | 49.4 (9.6) | 0.6975^#^ |
| Body mass index (BMI); median (IQR) | 26 (23-28) | NA | 25 (23-27) | 0.1454* |
| CD4 count (cells/µL); median (IQR) | 605 (490-777) | 360 (240-435) | NA | <0.0001* |
| CD8 count (cells/µL); median (IQR) | 610 (450-965) | 675 (337-977) | NA | 0.9129* |
| CD4:CD8 ratio; median (IQR) | 0.96 (0.69-1.35) | 0.47 (0.22-0.79) | NA | 0.0002* |
| Years known HIV^+^ status; median (IQR) | 12 (9-20) | 1 (1-2) | NA | <0.0001* |
| Duration of suppressive therapy; median (IQR) | 7 (6-13) | NA | NA | ND |
| Treatment Regimen, *N* (%)  3TC/DTG  ABC/3TC/DRV/ritonavir  ABC/3TC/DRV/Cob  ABC/3TC/DTG  ABC/3TC/EFV  ABC/3TC/NVP  ABC/3TC/RPV  CAB/RPV*  DRV/COB/DTG  RAL/3TC/EFV  TAF/FTC/DTG  TAF/FTC/EFV  TDF/FTC/DTG  TDF/FTC/EFV  TDF/FTC/EVG/Cob  TDF/FTC/RPV | 1 (1.6)  3 (4.7)  1 (1.6)  26 (40.6)  5 (7.8)  2 (3.1)  4 (6.2)  1 (1.6)  1 (1.6)  1 (1.6)  6 (9.3)  1 (1.6)  3 (4.7)  3 (4.7)  1 (1.6)  5 (7.8) | NA | NA | ND |
| **Initiation of treatment** |  |  |  |  |
| CD4 count at treatment initiation (cells/µL); median (IQR) | 316 (140-405) | NA | NA | ND |

*3TC, Lamivudine; ABC, Abacavir; CAB/RPV*, Cabotegravir given together with rilpivirine intramuscularly as long-acting drugs; Cob, Cobistat; DRV, Darunavir; DTG, Dolutegravir; EFV Efavirenz; EVG, Elvitegravir; FTC, Emtricitabine; N, Number; NA, Not Available; ND, Not Done; NVP, Nevirapine; RAL, Raltegravir; RPV, Rilpivirine; TAF, Tenofovir Disoproxil; TDF, Tenofovir Alafenamide; ^+^ Chi-square test, *Mann-Whitney U-test, # One-way ANOVA*

**Supplementary Table 2:** Clinical characteristics of longitudinal cohort

| **Parameter** | **PLWH_ART_** |
| --- | --- |
| *N* | 11 |
| Gender, Female, *N* (%) | 2 (18.2) |
| **At initiation of therapy** | |
| Age in years, mean (SD) | 43 (9) |
| CD4 count (cells/µL); median (IQR) | 300 (270-350) |
| CD8 count (cells/µL); median (IQR) | 600 (520-856) |
| CD4:CD8 ratio; median (IQR) | 0.47 (0.34-1.11) |
| **At sampling during cART** | |
| Age in years, mean (SD) | 52 (9) |
| CD4 count (cells/µL); median (IQR) | 690 (430-760) |
| CD8 count (cells/µL); median (IQR) | 510 (370-700) |
| CD4:CD8 ratio; median (IQR) | 0.99 (0.83-1.89) |
| Duration of suppressive therapy; median (IQR) | 8 (6-8) |
| Treatment Regimen, *N* (%)  ABC/3TC/DTG  TAF/FTC/DTG  TDF/FTC/EFV  TDF/FTC/RPV | 5 (45.5)  2 (18)  2 (18)  2 (18) |

*3TC, Lamivudine; ABC, Abacavir; DTG, Dolutegravir; EFV Efavirenz; FTC, Emtricitabine; N, Number; NA, Not Available; ND, Not Done;; RPV, Rilpivirine; TAF, Tenofovir Disoproxil; TDF, Tenofovir Alafenamide;*

**Supplementary Table 3:** Samples used per experimental method in this study

| **Experimental method** | **HC** | **PLWH_ART_** | **PLWH_VP_** |
| --- | --- | --- | --- |
| Untargeted metabolomics, *N* | 22 | 29 | 11 |
| Targeted metabolomics, *N* | 37 | 55 | 24 |
| OLINK inflammatory panel, *N* | 37 | 55 | 24 |
| Flow cytometry; metabolite transporters, *N* | 9 | 27 | - |
| Flow cytometry; myeloid cell lineages, *N* | 10 | 29 | - |
| Isolation of cell subsets, *N* | 10 | 29 | - |
| Intracellular measurement in isolated CD4^+^ T cells, *N* | 8 | 28 | - |
| Intracellular measurement in isolated monocytes, *N* | 6 | 27 | - |

**Supplementary Table 4:** List of antibodies

| **Protein** | **Cat no#** | **Company** |
| --- | --- | --- |
| FITC anti-CD3 (UCHT1) | 300406 | Biolegend |
| PE anti-CD4 (OKT4) | 317410 | Biolegend |
| APC anti-CD8 (RPA-T8) | 301038 | Biolegend |
| BV510 anti-CD14 (M5E2) | 301842 | Biolegend |
| BV785 anti-CD16 (3G8) | 56390 | BD Bioscience |
| BV711 anti-CD3 (OKT3) | 317328 | Biolegend |
| BUV395 anti-CD4 (SK3) | 563552 | BD Bioscience |
| APC anti-CD8 (RPA-T8) | 301014 | Biolegend |
| BV510 anti-CD14 (M5E2) | 301742 | Biolegend |
| BV786 anti-CD16 (3G8) | 563690 | BD Bioscience |
| FITC anti-Glut1 (202915) | FAB1418-F | R&D Systems |
| AF594 anti-xCT | NB300-318AF594 | Novus Biologicals |
| AF405 anti-MCT-1 (#82616) | FAB8275V | R&D Systems |
| PE-Cy7 anti-HLADR (L243) | 335830 | BD Bioscience |
| PE-CF594 anti-CD3 (UCHT1) | 562280 | BD Bioscience |
| PE-CF594 anti-CD19 (HIB19) | 562294 | BD Bioscience |
| PE-CF594 anti-CD56 (B159) | 562289 | BD Bioscience |
| FITC anti-CD33 (WM-53) | 564588 | BD Bioscience |
| BUV737 anti-CD11c (B-LY6) | 741827 | BD Bioscience |
| PE anti-CD14 (M5E2) | 555398 | BD Bioscience |
| APC-H7 anti-CD16 (3G8) | 560195 | BD Bioscience |
| BV421 anti-CD1c (F10/21A3) | 565050 | BD Bioscience |
| BV711 anti-CD141 (1A4) | 563155 | BD Bioscience |
| BV786 anti-CD303 (V24-785) | 748000 | BD Bioscience |
| BV650 anti-CCR2 (1D9) | 747849 | BD Bioscience |
| PE-Cy5 anti-CCR5 (2D7) | 556889 | BD Bioscience |
| BV605 anti-CX3CR1 (2A9-1) | 744488 | BD Bioscience |
| BUV395 anti-CD11b (ICRF44) | 563839 | BD Bioscience |
| AF700 anti-CD15 (HI98) | 301920 | Biolegend |
| AF647 anti-CD66b (G10F5) | 561645 | BD Bioscience |

**
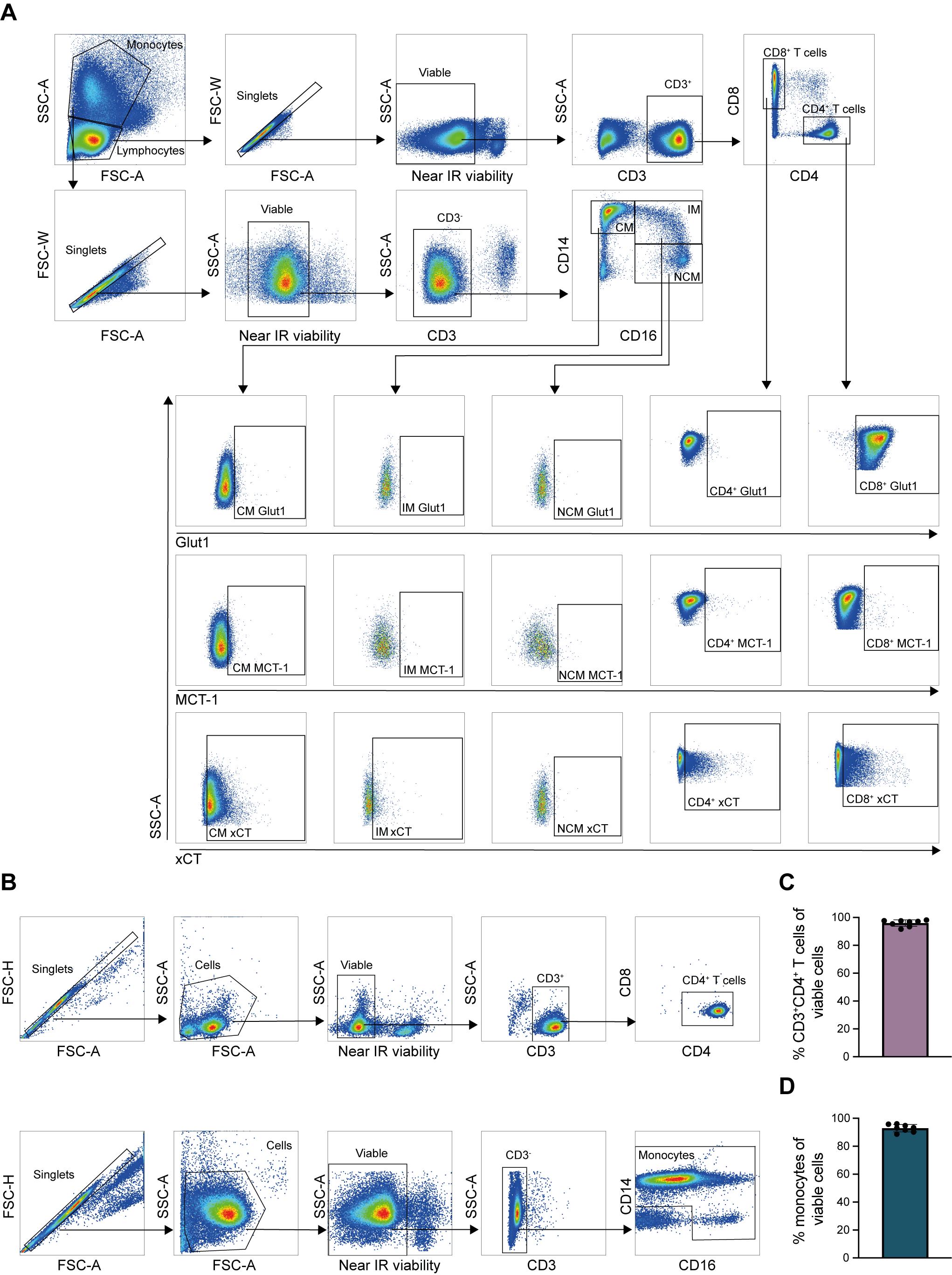
**

**Supplementary Figure 1:** Flow cytometry analysis of patient material. Related to Figure 3. (**A**) Gating strategy for metabolite transporters Glut1, MCT-1, and xCT in CD4^+^ and CD8^+^ T cells, CM, IM, and NCM. (**B**) Gating strategy verifying purity of isolated cell populations of CD4^+^ T cells and monocytes after isolation on EasySep columns. (**C**) Purity of CD4^+^ T cells (*n=8*). (**D**) Purity of monocytes (*n=8*). Statistical significance was determined using Mann-Whitney U-test (p<0.05) and represented with median and IQR.

**
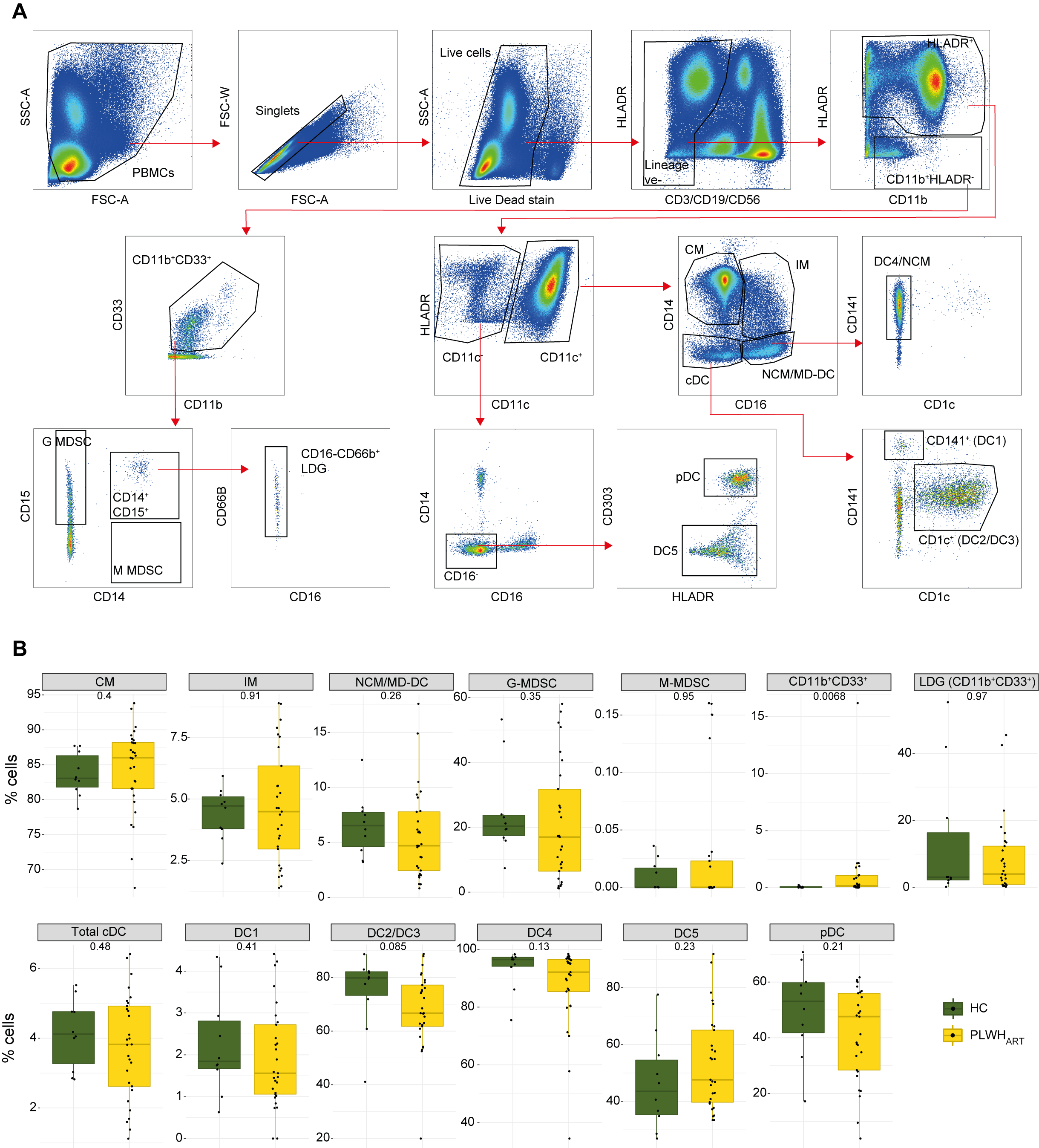
 Supplementary Figure 2:** Analysis of myeloid lineages in HC (*n=10*) and PLWH_ART_ (*n=29*). Related to Figure 4. (**A**) Gating strategy identifying monocytes, dendritic cells (DCs), and myeloid-derived suppressor cells (MDSCs). (**B**) Proportion of classical monocytes (CM), intermediate monocytes (IM), non-classical monocytes (NCM), granulocytic- myeloid derived suppressor cells (G-MDSC), mononuclear-myeloid derived suppressor cells (M-MDSC), low density granulocytes (LDG), classical dendritic cells (cDC), dendritic cells (DC)1/2/3/4/5, and plasmacytoid dendritic cell (pDC) populations in %. Statistics were performed using Mann-Whitney U-test (p<0.05) and visualized using median and IQR. Abbreviations:

**
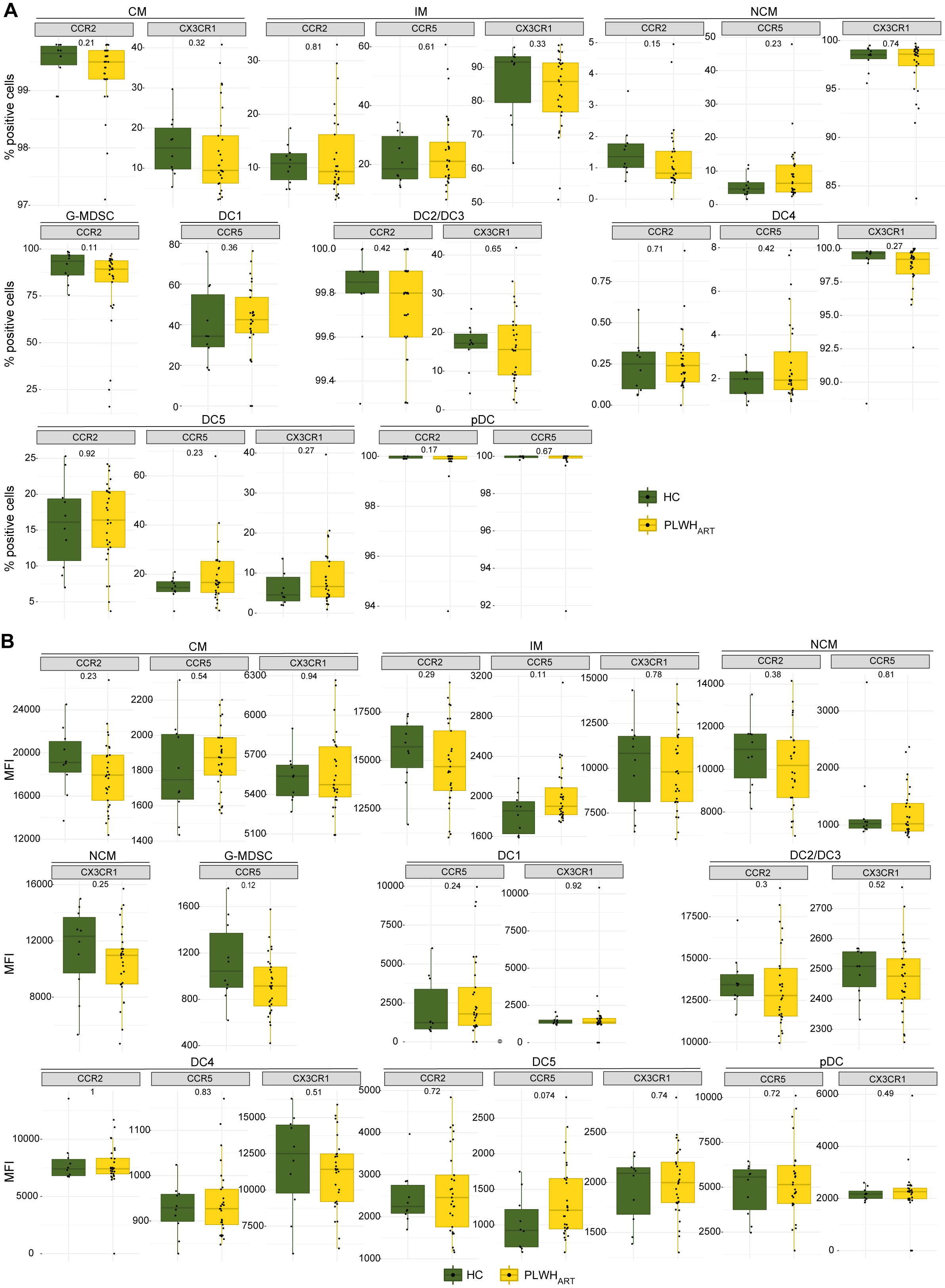
Supplementary Figure 3:** Cell receptor expression of CCR2, CCR5, and CX3CR1 in HC (*n=10*) and PLWH_ART_ (*n=29*). Related to Figure 4. (**A**) Proportion of cells expressing CCR2, CCR5, and CX3CR1 in all myeloid subpopulations in %. (**B**) Median fluorescence intensity (MFI) of CCR2, CCR5, and CX3CR1 expression in cell populations from the myeloid lineage in %. Statistics were performed using Mann-Whitney U-test (p<0.05) and visualized using median and IQR.
